# Signaling Pathways Regulating Redox Balance in Cancer Metabolism

**DOI:** 10.3389/fonc.2018.00126

**Published:** 2018-04-23

**Authors:** Maria Chiara De Santis, Paolo Ettore Porporato, Miriam Martini, Andrea Morandi

**Affiliations:** ^1^Department of Molecular Biotechnology and Health Science, Molecular Biotechnology Center, University of Torino, Torino, Italy; ^2^Department of Experimental and Clinical Biomedical Sciences, University of Florence, Florence, Italy

**Keywords:** metabolic reprogramming, one-carbon metabolism, OXPHOS, oncometabolites, pentose phosphate pathway

## Abstract

The interplay between rewiring tumor metabolism and oncogenic driver mutations is only beginning to be appreciated. Metabolic deregulation has been described for decades as a bystander effect of genomic aberrations. However, for the biology of malignant cells, metabolic reprogramming is essential to tackle a harsh environment, including nutrient deprivation, reactive oxygen species production, and oxygen withdrawal. Besides the well-investigated glycolytic metabolism, it is emerging that several other metabolic fluxes are relevant for tumorigenesis in supporting redox balance, most notably pentose phosphate pathway, folate, and mitochondrial metabolism. The relationship between metabolic rewiring and mutant genes is still unclear and, therefore, we will discuss how metabolic needs and oncogene mutations influence each other to satisfy cancer cells’ demands. Mutations in oncogenes, i.e., PI3K/AKT/mTOR, RAS pathway, and MYC, and tumor suppressors, i.e., p53 and liver kinase B1, result in metabolic flexibility and may influence response to therapy. Since metabolic rewiring is shaped by oncogenic driver mutations, understanding how specific alterations in signaling pathways affect different metabolic fluxes will be instrumental for the development of novel targeted therapies. In the era of personalized medicine, the combination of driver mutations, metabolite levels, and tissue of origins will pave the way to innovative therapeutic interventions.

## Introduction: Metabolic Deregulation and Tumor Progression

Despite being considered for decades as a bystander effect of the genomic alterations that characterize a cancer cell, it is now established that the deregulation of key metabolic hubs can be a tumorigenic driver and that several cellular signaling alterations converge toward metabolic alterations and the accumulation of specific intermediates endowed with oncogenic potential (1). The altered metabolic scenario of a cancer cell inevitably affects its cellular redox homeostasis. Particularly, cancer cells are characterized by an altered redox status and enhanced reactive oxygen species (ROS) that have been shown to drive and sustain cancer cells proliferation. Higher ROS levels are compensated by an increase in antioxidant mechanism that allows the cancer cell to survive in a pro-oxidant environment. However, such pro-oxidant condition favors DNA damage and genomic instability, events that concur to enhance the malignant traits of cancer cells, including metabolic reprogramming. Metabolic reprogramming and redox homeostasis are intimately interconnected and, therefore, such a vicious loop is further promoted by genetic lesions that activate proto-oncogenes or repress onco-suppressors, leading to cancer progression (2).

This review gathers the recent findings on the role of oncogenic-dependent metabolic reprogramming that cancer cells undergo during the different stages of tumor progression. Importantly, the main metabolic pathways concurring to redox homeostasis have been reviewed with a focus on the correlation between oncogenic lesions and ROS-dependent metabolic reprogramming.

## Pentose Phosphate Pathway (PPP)

The PPP, also termed phosphogluconate pathway, diverges from the glycolytic pathway after the initial phosphorylation of glucose catalyzed by the glycolytic enzyme hexokinase. Glucose-6-phosphate (G6P) is the principal substrate of the G6P dehydrogenase (G6PDH), the rate-limiting enzyme of the PPP. The primary endpoint of the PPP is to provide (i) phospho-pentoses, for the nucleotides and nucleic acids synthesis and (ii) reducing equivalents that are used for both reductive biosynthesis reactions and for redox homeostasis. Indeed, the production of reduced nicotinamide adenine dinucleotide phosphate (NADPH) in the oxidative phase of the pathway is essential for redox cellular homeostasis; directly, by buffering enhanced reactive oxygen intermediates and indirectly for the regeneration of the oxidized form of the glutathione (GSH), a pivotal molecule in neutralizing intracellular ROS levels (3). The non-oxidative phase of the PPP is characterized by a series of reversible reactions catalyzed by transaldolase (TALDO) and transketolase (TKT) enzymes that recruit additional glycolytic intermediates, i.e., fructose-6-phosphate (F6P) and glyceraldehyde-3-phosphate (G3P), to produce phospho-pentoses (4).

Despite leading to glucose oxidation, the main role of the PPP is anabolic rather than catabolic. Depending on the cellular requirements and response to exogenous stimuli, the PPP and the glycolysis cooperate to provide the needed metabolites. For instance, rapidly proliferating cells will boost the PPP to meet the requirements for pentoses to support nucleotide biosynthesis, both from G6P (oxidative phase) and from F6P and G3P (non-oxidative phase). While this condition is representative of a rapidly dividing cancer cell, a cell that has to maintain the redox cellular balance will sustain the oxidative branch for NADPH production and re-direct the non-oxidative branch toward F6P synthesis from the phospho-pentoses, which are then regenerating G6P to replenish the oxidative branch. ROS accumulation and subsequent oxidative stress result from the unbalance between ROS generation (e.g., from electron transport chain), and antioxidant mechanism (e.g., GSH, thioredoxin, and catalase) requiring NADPH to function as ROS scavengers. Therefore, not surprisingly, PPP deregulation has been linked to the pathogenesis of several diseases, such as G6PDH-deficiency. G6PDH-deficiency, and subsequent reduced activity, impairs the ability of erythrocytes to generate NADPH, hence exposing the cells’ phospholipid bilayer to the detrimental effect of ROS, leading to hemolytic anemia. G6PDH-deficiency is not associated with acquired susceptibility to particular diseases and preventing oxidative stress-inducing situations (i.e., certain drugs and food) leaves G6PDH-deficiency bearing individuals asymptomatic. Interestingly, G6PDH-deficiency is protective against malaria, heart, and cerebrovascular disease (5).

However, since the focus of this review is on cancer, we will discuss how PPP deregulation is affected by oncogenic-dependent metabolic reprogramming and how this impacts on tumor progression and therapy resistance. Interestingly, G6PDH-deficiency has been reported to reduce cancer susceptibility and incidence. A retrospective observational study in ~4,000 patients that underwent colonoscopy with a 10 years follow-up from Sardinia region (Italy), where G6PDH-deficiency prevalence ranges between 12 and 24% and is often caused by the G6PDH^C563T^ variant, shows that G6PDH-deficiency is associated with reduced colorectal cancer risk (5, 6). The potential explanation of such counterintuitive epidemiological data may be explained by the NADPH and PPP dependence that sustain lipid anabolism in cancer cells. Additionally, NADPH deficiency due to G6PDH alterations may alter the redox balance, hence reducing intracellular ROS levels, which play an important role in cancer initiation by increasing the DNA mutation rate and the synthesis of proinflammatory cytokines (7).

**Figure 1:**
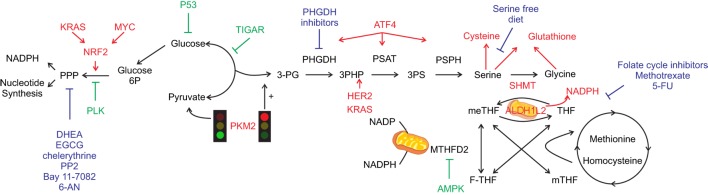
One-carbon metabolism (1-CM) and pentose phosphate pathway (PPP) at the cross-road between anabolism and redox balance. Simplified scheme of the crosstalk between metabolic and signaling pathways that can occur in cancer cells. Pro-tumorigenic signals are represented in red and anti-tumorigenic in green. Glycolysis regulates both PPP and 1-CM. Serine signaling pathway, once PKM2 activity is inhibited (red light), leads to glycolytic intermediates accumulation hence fueling both PPP and 1-CM. Serine metabolism enzymes bridge the shunt between glycolysis and 1-CM, with specific oncogene upregulating the enzymes involved [i.e., phosphoglycerate dehydrogenase (PGHDH), phosphoserine aminotransferase 1 (PSAT1), phosphoserine phosphatase (PSPH)], leading to serine generation and antioxidant machinery intermediates (cysteine and glutathione). Serine hydroxymethyltransferase (SHMT) mediates the conversion between serine and glycine with the concurrent transformation of tetrahydrofolate (THF) into 5,10-methylenetetrahydrofolate (meTHF). Aldehyde dehydrogenase 1 family member (ALDH1L) mediates the opposite reaction with concurrent generation of NADPH, similarly methylenetetrahydrofolate dehydrogenase (MTHDF) transform meTHF into 10-formyltetrahydrofolate (F-THF) with concurrent generation of nicotinamide adenine dinucleotide phosphate. The main chemotherapy agents are represented in blue. Dehydroepiandrosterone (DHEA), epigallocatechin gallate (EGCG), amino-5-(4-chlorophenyl)-7-(t-butyl)pyrazolo[3,4-d]pyrimidine (PP2), 6-aminonicotinamide (6-AN), and 5-fluorouracil (5-FU).

Glucose-6-phosphate dehydrogenase expression and activity have been proposed to be importantly influenced by genetic alterations that occur on oncogenes and/or oncosuppressor that ultimately lead to a pro-mitogenic signaling pathways activation of the cancer cells (Figure 1). Indeed, while the reprogramming of certain metabolic hubs seems to correlate with particular tumor phenotype and be associated with certain type of therapeutic option, the increased demand of NADPH, used for either antioxidant response and/or cellular anabolism than pentoses, is a prerequisite of almost any cancer (4). G6PDH is regulated by a plethora of extracellular stimuli, e.g., growth factors, that impacts on its expression and activity *via* the MAPK and PI3K signaling pathways. Since these signaling pathways are often hyper-activated in cancer due to oncogenes activation (e.g., K-RAS, MYC, and growth factor receptors), or oncosuppressors inactivation (e.g., p53 and PTEN loss-of-function and inactivation), any alterations that impact on these players may lead to G6PDH enhanced expression and activity (8, 9). Additionally, some of the aforementioned cancer-inducing genes can also regulate G6PDH function independently of the signaling cascade. For instance, loss of p53, which directly controls and inhibits glucose transporters expression (10), leads to enhanced glucose uptake that can be diverted into the PPP pathway. Similarly, glucose diversion into the PPP can also be a consequence of the downregulation of TP53-induced glycolysis and apoptosis regulator (TIGAR), a p53-dependent gene known to inhibit the glycolytic enzyme phosphoglycerate mutase 1 (PGAM1) that leads to the increase of the oxidative PPP by reducing the amount of 3-phosphoglycerate (3PG), which has been reported to inhibit the PPP enzyme 6-phosphogluconate dehydrogenase (6PGDH) (11). Finally, p53-mediated G6PDH inhibition can also be mediated by direct protein-to-protein contact (3). DNA damage, telomeric instability, or oxidative stress can activate the ataxia telangiectasia-mutated (ATM) kinase, which in turn activates p53. Interestingly, ATM promotes the Hsp27 phosphorylation and binding to G6PDH, stimulating its activity and, therefore, increasing the PPP flux and the subsequent NADPH and phospho-pentoses production (12). However, others have reported that loss of p53 enhances NADPH production, therefore, by generating a negative feedback on the PPP. These reported contradictory results may be related to the different role that p53 can exert on either cell cycle or apoptosis (3). Finally, it has been recently demonstrated that polo-like kinase 1 (PLK1), a key player in cell mitosis, is able to directly phosphorylate and activate G6PDH (13).

Although G6PDH is the major rate-limiting step of the PPP, the amount of phosphorylated glucose trapped into the cells and the expression of enzymes regulating the glycolytic rate can also affect the PPP flux. Indeed, higher levels of G6P are required to sustain oxidative and non-oxidative branches of the PPP and this can be achieved by (i) enhancing the amount of glucose that is phosphorylated by the glycolytic-rate limiting enzyme hexokinase 2 (HK2), the main isoform expressed by cancer cells, and (ii) by engulfing the glycolytic pathway and subsequent enhancing the accumulation of glycolytic intermediates. HK2 enhanced expression, known to be regulated by oncogenic RAS activation, is essential to sustain the non-oxidative phase of the PPP in lung cancer (14). Additionally, pyruvate kinase (PK)-M2, an isoform of the PK that converts phosphoenolpyruvate (PEP) into pyruvate and that is expressed by many cancers, can be controlled by post-translational modifications (e.g., oxidation, phosphorylation, and acetylation) that impair the PKM2 tetrameric-mediated metabolic function favoring the PKM2 dimer formation (15, 16). Particularly, an increase in the PKM2 dimer content induces an accumulation of the glycolytic intermediates, including that of G6P, that results in increased metabolic flux into the PPP and enhanced NADPH production (17–20).

6-Phosphogluconate dehydrogenase, the third enzyme involved in PPP, that catalyzes the oxidative decarboxylation of 6-phosphogluconate to ribulose 5-phosphate and CO_2_, with concomitant reduction of NADP to NADPH, has also been reported to be upregulated in many solid cancers and has been often correlated to G6PDH (4).

Transketolase and TALDO are the two key enzymes in the non-oxidative branch and divert glycolytic intermediates (e.g., F6P and G3P) into the PPP to fuel ribonucleotides biosynthesis, essential for fast proliferating cancer cells (21). Their expression levels have been found deregulated in cancer cells. Particularly, oncogenes activation that leads to hyper-proliferation may also have an impact on the non-oxidative branch of the PPP, favoring ribonucleotides biosynthesis. Indeed, the use of isogenic colorectal cancer cell lines that express either the mutant or the wild-type form of K-RAS or B-RAF shows that the enzymes of the non-oxidative branch of the PPP (ribose-5-phosphate isomerase and TKT) are upregulated in the K-RAS-mutant cell lines (20). These results are in line with a previous report showing that K-RAS mutations in pancreatic cancers enhance the non-oxidative branch of the PPP (22).

Due to the important role in maintaining the redox homeostasis, PPP is also importantly regulated by nuclear factor erythroid 2-related factor 2 (NRF2), a transcription factor that has an essential role in combating enhanced ROS levels and controlling ROS detoxification and homeostasis. NRF2, which is usually bound in its inactive form to the cytosolic Kelch-like ECH-associated protein 1 (KEAP1), is able to translocate into the nucleus upon NRF2-KEAP1 destabilization, oxidative stress, or protein succination driven by fumarate accumulation (23, 24). Once into the nucleus, NRF2 activates antioxidant response genes and this activation have been reported to play a major role in protecting cancer cells from the oxidative stress induced by antitumoral therapies. Activation of NRF2 or destabilization of NRF2–KEAP1 interaction caused by genetic modifications of NRF2 and KEAP1 has been reported in several cancer types, including those of liver, esophagus, intestine, lung, and breast (25, 26). Moreover, the increased expression of NRF2-dependent genes correlates with poor prognosis (27–29). NRF2 is tightly linked to metabolic reprogramming. In particular, NRF2 targeting impairs the activity and the expression of the enzymes involved in the PPP (e.g., G6PDH, TKT, and 6PGDH), a process that has been shown to be mediated by microRNAs (30). Recently, it has been shown that NRF2 enhances expression of the PPP enzymes. The consequent increase of the PPP flux leads to acquired proliferative advantage in the presence of constitutive activation of PI3K-AKT signaling pathway due to *PTEN* deletion (31). Oncogenic activation of K-RAS and B-RAF and overexpression of MYC, together with enhanced activation of the PI3K/AKT pathway induce NRF2 nuclear translocation, further reinforcing the link between oncogenes activation and NRF2-mediated PPP induction and redox homeostasis alterations.

An important driver of cancer cell growth and survival is the activation of the PI3K/AKT pathway that can occur as a consequence of external stimuli, e.g., growth factor receptor activation or as a consequence of a genetic lesion, e.g., *via* PTEN loss-of-function that ultimately leads to PI3K/AKT-mediated mTOR activation. It has been extensively reported that mTOR can control cell metabolism, including the glycolytic pathway and PPP (32). mTOR kinase can associate with different subunits leading to two signaling complexes, mTORC1 and mTORC2. Particularly, mTORC1 regulates cell proliferation and anabolism.

Indeed, it has been shown that mTORC1 activation sustains the metabolic flux through both glycolysis and the oxidative arm of PPP. Duvel and coworkers demonstrated that mTORC1 activation promotes G6PDH expression, a process that is in part mediated by SREBP1 (33). Additionally, it has been recently reported that mTOR-dependent G6PDH expression can be controlled by androgen receptor signaling in prostate cancer models (34). In the Duvel et al. manuscript it was also reported that mTOR activation induces HIF-1α expression and stabilization, leading to the transcriptional activation of a plethora of metabolic HIF-dependent genes in a hypoxia-independent mechanism (33). HIF-1α stabilization have been also reported to control the expression of TKT in pancreatic cancer cells, hence impacting on the non-oxidative branch of the PPP (35) and subsequent response and resistance to gemcitabine. However, also hypoxia has been reported to impact on PPP flux. Indeed, colon cancer cells subjected to 1% hypoxia increase their intracellular levels of ribose-phosphates and gluconic-acid, terminal and intermediate compounds of the oxidative branch of PPP, respectively (36).

## One-Carbon Metabolism (1-CM): The Serine Synthesis Pathway (SSP)

One-carbon (1-C) metabolism is responsible for the transfer of 1-C unit through folate intermediates, coupling the folate and the methionine cycle. This pathway is required for nucleic acid synthesis (purine and thymidine), amino acids homeostasis (methionine, serine, and glycine), antioxidant defense (NADPH production) and epigenetic maintenance (homocysteine re-methylation) (37–39). In particular, the methionine cycle produces the substrate for the S-adenosyl methionine (SAM)-dependent methyl transferases. These enzymes are responsible for the addition of methyl groups to proteins, lipids, secondary metabolites, and nucleic acids and are, therefore, essential for epigenetic modifications.

In order to sustain the NADPH-mediated antioxidant defense, 1-CM supplements the major source of intracellular NADPH, which is produced by the oxidative branch of the PPP and by the malic enzyme. In the 1-C cycle, there are two steps that can lead to NADPH production: one catalyzed by the methyl dehydrogenases, mitochondrial 2-like (MTHFD2L) and cytosol MTHFD1, and the other catalyzed by the formyl dehydrogenases, cytosolic aldehyde dehydrogenase 1 family member L1 (ALDH1L1) and mitochondrial aldehyde dehydrogenase 1 family member L2 (ALDH1L2).

1-C units are mainly derived from serine, formate, and histidine, which are directly used in the cytosolic folate cycle or by glycine, sarcosine, and dimethylglycine, which are converted into folate and secreted into the cytosol (40, 41). Glycine has a prominent role because it can contribute to both the folate cycle and to serine production. In particular, the folate cycle is completed in the cytosol by serine hydroxymethyltransferase 2 (SHMT2), which catalyzes the concomitant conversion of l-serine to glycine and tetrahydrofolate to 5,10-methyleneTHF. Hence, glycine represents a precursor of glutathione and purines, required for antioxidant defense and proliferation, respectively. If necessary, glycine can be converted into serine with the concomitant production of 5,10-methylene-THF, although some of the specificity of serine-related metabolic functions are lost and this results, for instance, in decreased purine synthesis and reduced cell proliferation (42, 43).

The main donor of 1-C to the folate cycle is the non-essential amino acid serine that can be synthetized *de novo* by the cell (41), a process that requires three MYC-regulated enzymes, PHGDH, PSAT1, and PSPH. The backbone of serine is derived from the glycolytic or gluconeogenic pathway, with the production of the intermediate 3PG. The main transcriptional activator of the three SSP enzymes is ATF4, a cAMP-response element-mediated transcription factor that mediates oxidative stress response (*via* NRF2) (44), serine starvation (*via* mouse double minute 2 homolog MDM2) (45), and histone methylation (41).

At physiological levels, serine can act as an allosteric activator of the metabolic activity of PKM2, which catalyzes the last step of glycolysis, preventing the redirection of 3PG into serine synthesis and promoting glycolysis. Similarly, AKT-mediated phosphorylation of MDM2, a negative regulator of p53, on Ser166 induces the association of MDM2 to PKM2, in order to promote PKM2 activity. In condition of oxidative stress, serine deprivation, and the consequent decrease in the allosteric activation of PKM2, the non-phosphorylated form of MDM2 is recruited to the chromatin with ATF4, which activates a transcriptional program involved in amino acid metabolism and redox homeostasis (45–48). In particular, during oxidative stress, ATF4 has been reported to be transcriptionally activated by NRF2, which induces the transcription of SSP-related genes and regulates the antioxidant response (44). While during serine starvation, the inhibition of PKM2 metabolic activity leads to the accumulation of glycolytic intermediates that results in *de novo* serine synthesis activation, if G3P is used as a substrate for the PHGDH, or into oxidative PPP activation and/or if G6P is used as a substrate for G6PDH, as described above (27).

mTORC1 signaling, through ATF4, activates the transcription of MTHFD2 in both normal and cancer cells, increasing *de novo* purine synthesis necessary for nucleic acid production (48). PKM2-expressing cells can maintain mTORC1 activity and proliferate in serine-depleted medium (49).

It has been reported that highly proliferative cells require an exogenous amount of serine supply for their optimal growth to adjunct the *de novo* synthesis (50). Indeed, additional serine sources are derived from diet intake, from breakdown of intracellular proteins, and from the conversion of glycine. In serine starve condition, P53 null cells have an impaired proliferation rate and tumor growth is reduced in mice fed with serine-free diet (50).

Due to its role in maintaining amino acid and redox homeostasis and epigenetic regulation, it is not surprising that the SSP has been found altered in cancer. Since serine can become a limiting factor, cancer cells can adopt two strategies to bypass this issue: they can increase *de novo* serine synthesis to become dependent on some alternative amino acids, such as glutamine, or can promote exogenous serine intake (41). For instance, PHGDH is commonly altered in cancer cells, either upon gene amplification (51, 52), or activation of transcription factors promoting expression of SSP enzymes, e.g., MYC and NFR2 (44). Among the signaling pathways that promote the 1-C metabolism, it has been reported that HER2 amplification (53) increases the expression of PHGDH (Figure 1). The PHGDH promoter is positively regulated by specificity protein 1 (SP1) and nuclear transcription factor Y (NFY), two transcription factors that are often upregulated in cancer (54). PHGDH is overexpressed and associated with poor prognosis in triple negative breast cancer and melanoma (44, 52, 55). While genomic alterations are typical of PHGDH, the high expression of the folate enzyme is due to aberrant transcriptional regulation. In non-small cell lung cancer (NSCLC), ATF4 overexpression can be caused by KEAP1-mediated activation of NRF2 (44) or activation of the PI3K/mTOR signaling pathway (48, 56, 57).

Under hypoxia, HIF-1α and MYC can mediate SHMT2 increased levels that correlate with poor prognosis. Among the enzymes that catalyze the reactions coupled with the production of NADPH, THF dehydrogenases (cytosolic ALDH1L1 and mitochondrial ALDH1L2) and MTHF-dehydrogenases (mitochondria MTHFD2L and cytosolic MTHFD1) are involved in cancer. ALDH1L1 is usually underexpressed in cancer cells (57), as its overexpression would deplete the cytosolic 10-formyl-THF used for the synthesis of nucleotides. Conversely, ALDH1L2 is overexpressed to control mitochondrial redox homeostasis under hypoxia and to support melanoma cells metastatization (56). K-RAS activating mutation is associated with the high expression of the folate metabolism enzyme, MTHFD2 (47), and this leads to increased nucleotide synthesis, increased ATP and NADH production, and finally to enhanced mitochondrial NADPH production which is essential for struggling ROS increased levels. Conversely, during nutrient deprivation or hypoxia, when intracellular levels of ATP decline, 5′ AMP activated kinase (AMPK) activation represses the expression of MTHFD2 (50, 58).

## Mitochondrial Metabolism

Reactive oxygen species mtROS are generated by mitochondria as a natural by-product of electron transport chain activity. Recently, it has been reported that mitochondria ROS (mtROS) can activate tumorigenic signaling and metabolic reprogramming. Increased production of ROS has long been observed to be a hallmark of many tumors and cancer cell lines. Consequently, this oncogenic signaling increases the expression of antioxidant proteins to balance the high production of ROS to maintain redox homeostasis.

Indeed, several aspects of mitochondrial biology, including biogenesis and turnover, fission and fusion, and mitochondria-controlled metabolic signaling are controlling cellular transformation (59, 60).

Mitochondria are signaling hubs and bioenergetics organelles, which play an important role in cellular adaptation to environmental changes, directly responding to nutrient availability. For instance, during stress condition, such as extracellular acidosis or hypoxia (60, 61), mitochondria tune their metabolism to support fatty acid synthesis by upregulating the reductive carboxylation of α-ketoglutarate *via* citrate generation to support both production of lipid membranes and production of intermediates for protein acetylation (13).

An element controlling mitochondrial function is the mitochondrial mass, which can vary greatly in cell, depending on the microenvironmental context and oxidative fuels availability of certain nutrients. The mitochondrial mass is regulated by biogenesis and turnover, two different pathways that can act as positive and negative regulators of tumorigenesis (59). It is generally accepted that MYC is one of the key activator of mitochondrial biogenesis in cancer, and opposed effects are exerted by the activation of HIF-1α signaling pathway (62), as well as FOXO3a (62–64). In physiological conditions, MYC couples mitochondrial biogenesis with cell-cycle progression, whereas once deregulated in cancer it stimulates mitochondrial metabolism to support rapid cell growth (63). The major impact of MYC on mitochondria function depends on the direct regulation of the transcriptional coactivator peroxisome proliferator-activated receptor gamma coactivator-1 alpha (PPARGC1A, best known as PGC-1α) (58), that is responsible for the enhanced metabolic plasticity of aggressive cancer cells. In particular, PGC-1α levels have a dichotomous effect on tumors, with an upregulation that has been associated with pro- or anti-tumorigenic effects on the basis of tumor types and experimental conditions (65–67).

The discovery of the tumor suppressor liver kinase B1 (LKB1) and its major downstream effectors PGC-1α and AMPK established a central metabolic hub at the crossroad between energy regulation and cancer development (68). It has been already reported that LKB1 plays an important role in reducing intracellular ROS in response to oxidative stress (69, 70). Consequently, LKB1 loss increases oxidative DNA damage and mutations induced by the accumulation of ROS. In NSCLC, the redox imbalance caused by LKB1 inactivation modulates tumor plasticity and promotes tumor progression *via* metabolic adaptation (71).

In addition to aerobic glycolysis, cancer cells often rely on elevated glutaminolysis, supporting mitochondrial metabolism for cancer growth. In order to fuel the tricarboxcylic acid (TCA) cycle, glutamine is first converted to glutamate by glutaminase and then to α-ketoglutarate by glutamate dehydrogenase or aminotransferases. Therefore, glutamine supports the high proliferating rate of cancer cells by acting as substrate for the TCA cycle to produce building blocks, including ATP, lipids, nucleotides, and proteins. MYC oncogene transcriptionally promotes glutaminolysis and the use of glutamine as a bioenergetic substrate (72). The final effect of MYC-dependent glutaminolysis is a profound mitochondrial metabolism reprogramming due to glutamine catabolism dependency and TCA cycle anaplerosis. Noteworthy, reductive carboxylation under hypoxia has been shown to promote generation of 2-hydroxyglutarate (2HG) even in absence of isocitrate dehydrogenase (IDH) mutations (73, 74).

Recently, it has been reported that an association with the oncometabolite 2HG accumulations and MYC pathway activation in breast cancer patients (75). This analysis identified a subtype of tumors characterized by higher levels of 2HG that associate with poor prognosis and with a distinct DNA methylation pattern. These tumors tend to overexpress glutaminase, suggesting a functional relationship between MYC activation and glutamine dependence in breast cancer (75, 76).

2-Hydroxyglutarate is a TCA byproduct that can be generated in certain conditions and has been termed oncometabolite, since its production can drive oncogenesis (77). To date, various germline and somatic mutations in mitochondrial enzymes led to oncometabolites accumulation. For instance, succinate dehydrogenase (SDH), fumarate hydratase, and IDH (78) once mutated lead to cellular transformation and oncogenesis (79) *via* augmented levels of fumarate, succinate, and 2HG, respectively (79). Those molecules present various activity, altering epigenetic pattern, enzymatic activity, or the aforementioned protein succination induced by fumarate. Coherently with the oncogenic role of such metabolite, TRAP1 expression has been shown to induce cellular transformation by inhibiting SDH, thus promoting succinate build-up (80, 81).

Recently, Morita et al. reported a key role for mTORC1 signaling, stimulating mitochondrial biogenesis and activity, bolstering ATP production capacity (82). This effect is mediated by the activity of 4E-BP proteins that mediate mTORC1-driven translation of mitochondria-related mRNAs, mitochondrial respiration and biogenesis, and ATP production. These data reveal a feed-forward mechanism by which translation impacts mitochondrial function to maintain cellular energy homeostasis (82). Consistently, in muose Embryonic Stem cells has been shown that tumorigenicity and teratoma formation was bound to increased mitochondrial metabolism linked to higher mTOR activity (83).

**Figure 2:**
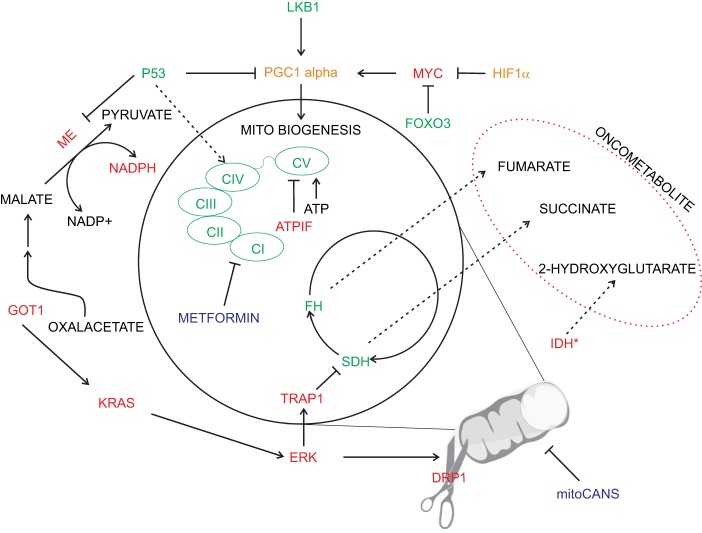
Oncogenic deregulation in mitochondrial metabolism. Physiological mitochondrial metabolism is deranged in cancer by several oncogenic signaling (depicted in red). Inhibition of specific mitochondrial enzymes [i.e., succinate dehydrogenase (SDH) and fumarate hydratase (FH)] or specific mutation [i.e., isocitrate dehydrogenase (IDH)] leads to cellular transformation *via* the generation of oncometabolites. Similarly, oncogenic drivers affect electron transport chain activity and promote increased oxidative stress buffering (e.g., *via* malic enzyme-ME). Pro-tumorigenic signals are represented in red and anti-tumorigenic in green. Enzymes with a double pro/anti-tumorigenic signal according to the type of tumor (i.e., PGC1 alpha and HIF1α) are represented in orange. The main chemotherapy agents are represented in blue.

Mitochondria are extremely dynamic structures and the balance of fission and fusion is responsible for their morphology (84). The mitochondrial division is controlled by dynamin-related protein 1 (Drp1), a GTPase that is recruited to the outer mitochondria membrane. Drp1 mitochondrial translocation is regulated by different kinases that are activated by specific cell-cycle and stress conditions. Several studies described that cancer cells have an imbalance fission-fusion state, characterized by high fission and decreased fusion activities, causing a fragmentation of mitochondrial network (85). Disruption of mitochondrial dynamics are one of the key features of K-RAS-induced cellular transformation, by stimulating mitochondrial fragmentation *via* ERK1/2-mediated phosphorylation of Drp1 (86). In addition, the mitochondrial remodeling caused by oncogenic K-RAS directly increases ROS generation and impacts the mitochondrial function itself (87). Similarly to what has been reported for the MYC oncogenic activation, the mitochondrial network remodeling caused by oncogenic K-RAS generates general tumorigenic stimuli that promote transformation. However, further studies are needed to understand the effects of oncogenic signaling pathways on mitochondrial dynamics. K-RAS-mediated metabolic remodeling drives also an augmented glutamine metabolism and malic enzyme activity, acting as an important source of NADPH (Figure 2) (88). Mitochondrial remodeling is also altered by ATPase inhibitory factor 1 (ATPIF), a factor exerting pro-tumorigenic function by preventing cristae remodeling and mitochondrial depolarization by inhibiting ATPase activity of complex V (89).

Redox homeostasis is an important process that can fuel anchorage-independent growth. In this stressful condition, mitochondrial IDH2 supports NADPH pool to decrease mtROS accumulation (41). Consequently, the limitation of excessive generation of mtROS is a converging pattern that can support metastasis formation, although a moderate degree of mtROS directly promotes metastasis formation (56, 90). Accordingly, inhibiting mitophagy by BNip3, hence degradation of dysfunctional mitochondria, promotes mtROS generation sufficient to induce metastasis in breast cancer (91).

Another important player in the regulation of mitochondrial metabolism and intracellular redox homeostasis is p53, a key regulator of several biological processes, including energy homeostasis, apoptosis, and cell-cycle arrest, through transcription-dependent and -independent mechanisms (92). To regulate energy metabolism, p53 represses glycolysis, by regulating the expression of TIGAR, PGAM1, and PDK2 (pyruvate dehydrogenase kinase 2), and promotes oxidative phosphorylation process.

Beside on its nuclear role for gene expression, a transcription-independent role of p53 in inducing mitochondrial apoptosis has been suggested (93). The mitochondrial regulation of p53 is critical for the control of the cellular switch between survival and apopotosis. In fact, several studies have shown that after DNA damage, p53 is translocated to mitochondrial outer membrane, causing mitochondrial outer membrane permeabilization (MOMP), caspase-3 activation, and cytochrome c release (94).

Altogether these studies demonstrate that mitochondria are crucial players of tumorigenesis, given that this process requires the ability to adapt to cellular and environmental alterations. Therefore, a deep understanding of molecular mechanisms regulating the function of mitochondria will be critical for the next generation of anti-cancer agents.

## Targeting Oncogene-Dependent Metabolism: Therapeutic Opportunities

One of the biggest challenges in cancer management is to ensure a tailored therapeutic approach to the patients that will lead to the administration to the right drug (as either monotherapy or combination), at the right patient in the right moment. Target therapies have expanded the portfolio of therapeutic opportunities and are often used either alone or as adjuvant therapies with chemotherapeutic agents. Cancer patients are usually matched to targeted therapies depending on driver mutations profiled in their tumors. However, the metabolic landscape of the tumor profoundly affects the response to therapy (95) and should be considered for optimal therapeutic intervention. Additionally, independently of the oncogenic drivers that sustain cancer growth and progression, understanding the metabolic network and the requirements of a given cancer, will offer a series of additional potential targets that could be exploited therapeutically.

For instance, since the PPP plays a key role in ribonucleotides biosynthesis, necessary for duplicating tumor cells, and redox homeostasis, essential to handle the oxidative stress induced by anti-tumoral therapies, targeting key step of the PPP has been proposed as a potential therapeutic opportunity. However, few inhibitors targeting PPP enzymes are available and no clinical trials have been designed to investigate PPP targeting in solid cancers. However, in the preclinical setting PPP targeting has been effective. Due to the rate-limiting role of the G6PDH enzyme, its targeting has been proposed and a series of compounds able to inhibit its activity *in vitro* and/or *in vivo* have been reported. Among them, the best characterized is the adrenal hormone dehydroepiandrosterone (DHEA), shown to be effective in inhibiting proliferation and survival of different cancer models (96, 97). To date, 60 trials are (or have been) investigating the application of DHEA in different cancer types (clinicaltrials.gov). However, the majority of the studies have evaluated DHEA for its hormonal effect rather than for its ability to target PPP. In the concluded trials, DHEA is overall well tolerated and further studies should be designed to draw any conclusion on its anti-metabolic effect; accordingly, patients should be subgrouped based on their metabolic dependency, with a particular focus on G6PDH expression. Other compounds have been reported to target G6PDH, such as epigallocatechin gallate (78), chelerythrine (initially a PKC inhibitor), Amino-5-(4-chlorophenyl)-7-(t-butyl)pyrazolo[3,4-d]pyrimidine (PP2) (98), and the Bay 11–7082 (99) but these compounds will require additional characterization in relevant cancer models prior to enter into the clinical setting. Interestingly, other compounds have been investigated in high-throughput screening leading to additional promising G6PDH inhibitors (100). The PPP can also be blocked by the 6-aminonicotinamide (6-AN) that promotes the biosynthesis of the 6-AN adenine dinucleotide phosphate which is able to inhibit the 6PGDH enzyme, hence reducing the NADPH production (4). In summary, targeting the PPP pathway may be a successful approach because, in addition to the reduction of the ribonucleotide biosynthesis that ultimately impacts on cell division, it could potentiate the effect of other therapies by reducing the resistance to the oxidative stress induced by anti-cancer compounds.

Importantly, for an emerging group of therapies, Mayers and colleagues have proposed that the metabolism of a target tumor may be a key determinant of its response and may be determined by the tumor’s tissue of origin and not exclusively by their mutation status [see the Perspective by Vousden and Yang (101)]. Indeed, branched-chain amino acids (BCAAs) destiny has been traced in lung and pancreatic cancer mouse models driven by the same mutations. Lung tumors showed increased BCAA nitrogen uptake and utilization for the biosynthesis of amino acid and nucleotides, while pancreatic tumors displayed decreased uptake of free BCAAs (102). These differences demonstrate that the tissue of origin shapes tumor metabolism and should be taken into account to ensure the optimal therapeutic approach.

It is established that p53 loss is a frequent aberration in many cancer cells and that one of the pro-tumoral effects of such genetic lesion is the increased serine dependency, indicating that limiting cellular serine availability may be a therapeutic option. Tumors with amplified SSP genes are only partially dependent on exogenous serine depletion and a potential therapeutic approach for this subset of cancer may come from impairing the *de novo* serine synthesis. For example, PHGDH inhibitors (21, 51, 103) could be useful in patients with PHGDH-amplified tumors (21, 51). Indeed, chemotherapy based on the inhibition of the folate metabolism and thymidylate synthesis has been the pioneer anti-cancer drugs that entered into the clinical management of cancer patients (104). For instance, methotrexate and 5-fluorouracil are still used in leukemias treatment, while pemetrexed, which acts by mimicking the folic acid and inhibiting the purines and pyrimidines biosynthesis, is administered to patients with mesothelioma and lung cancer (105, 106). Combination of a serine-free diet with metformin, an anti-diabetic biguanide that has been reported to target complex I of the electron transport chain among other targets, shows a synergistic antineoplastic effect (107). Side effects due to the inhibition of 1-CM in non-transformed cells mainly affect proliferating tissues, such as the intestinal epithelium and bone marrow, resulting in gastric damage, anemia, and immune deficiency. Circulating and intratumoral levels of serine and/or the expression of genes of the folate metabolism could be used as potential predictive biomarkers to identify subsets of cancers more likely to respond to anti-folate therapy (47, 108).

Mitochondria are gatekeepers of cell death and could, therefore, be considered as valuable targets for novel therapeutic avenues. The characteristic redox state of malignant cells, due to the altered electrochemical gradient across the inner mitochondrial membrane, renders mitochondria optimal target as shown by Leanza and coworkers (109). Metformin, as described above, has been recently proposed to be repurposed for cancer treatment in light of its activity as mitochondrial complex I inhibitor, promoting cancer specific cell death in a plethora of conditions, most notably in cancer stem cells (110, 111). In cancer tissues, the maintenance of mitochondrial structural integrity is essential to produce energy and overcome cellular stress, including nutrient deprivation and chemotherapy (112). Coherently, major efforts are ongoing for the development of novel molecules targeting directly targeting mitochondria in cancer (i.e., mitoCANS) (113).

## Concluding Remarks

Genomic aberrations play a major role in tumor development and targeting oncogenic drivers have been successful in many types of cancers. However, the advantages of the oncogenic activation in terms of enhancing proliferation and survival capacity, metastatic abilities and resistance to therapies have a profound effect on the metabolic network of a cancer. It is conceivable that, to prolong the efficacy of the current anti-tumoral therapeutic regimens or to combat the development of therapy resistance, a potential successful approach could be the targeting of the “engine” of the tumor growth, i.e., targeting its driving metabolism. Indeed, independently of the drivers that promote tumor progression and/or therapy resistance, metabolic reprogramming will occur, a process that has been proposed to converge according to the tissue of origin more than of the oncogenic drivers (114). Here, we have listed a series of metabolic pathways that are controlled by key oncogenes and oncosuppressors that are often deregulated in cancer and could be potentially targeted. The concomitant targeting of the drivers together with the engine (that sustain cancer growth and progression) will render the signaling and metabolic reprogramming more difficult to occur and could represent a valid therapeutic approach. Further preclinical investigations on synthetic lethality approach in different tumor types and on the potential side effects that such stringent combinatory approach could have on normal cells are required and, if successful, will represent a major contribution toward the introduction of the personalized therapy approach in cancer patients’ management.

## Author Contributions

MS, PP, MM, and AM selected the references and cowrote the text.

## Conflict of Interest Statement

The authors declare that the research was conducted in the absence of any commercial or financial relationships that could be construed as a potential conflict of interest.

## Abbreviations

1-CM, one-carbon metabolism; ALDH, aldehyde dehydrogenase 1 family member; αKG, alpha-ketoglutarate; DRP1, dynamin-related protein; F6P, fructose-6-phosphate; FH, fumarate hydratase; G3P, glyceraldehyde-3-phosphate; G6P, glucose-6-phosphate; G6PDH, glucose-6-phosphate dehydrogenase; IDH, isocitrate dehydrogenase; MDM2, mouse double minute 2 homolog; MOMP, mitochondrial outer membrane permeabilization; MTHFD, methylenetetrahydrofolate dehydrogenase (NADP + dependent); NADPH, nicotinamide adenine dinucleotide phosphate; OMM, outer mitochondria membrane; PGAM1, phosphoglycerate mutase 1; PGC-1α, peroxisome proliferator-activated receptor gamma coactivator-1 alpha; PHGDH, phosphoglycerate dehydrogenase; SDH, succinate dehydrogenase; SSP, serine synthesis pathway; TCA, tricarboxcylic acid; TIGAR, TP53-induced glycolysis and apoptosis regulator; PPP, pentose phosphate pathway; ROS, reactive oxygen species.
